# High-throughput SNP discovery through deep resequencing of a reduced representation library to anchor and orient scaffolds in the soybean whole genome sequence

**DOI:** 10.1186/1471-2164-11-38

**Published:** 2010-01-15

**Authors:** David L Hyten, Steven B Cannon, Qijian Song, Nathan Weeks, Edward W Fickus, Randy C Shoemaker, James E Specht, Andrew D Farmer, Gregory D May, Perry B Cregan

**Affiliations:** 1 Soybean Genomics and Improvement Laboratory, U.S. Department of Agriculture, Agricultural Research Service, Beltsville, MD 20705, USA; 2 Department of Agronomy, U.S. Department of Agriculture, Agricultural Research Service, Iowa State University, Ames, IA 50011, USA; 3 Department Plant Science and Landscape Architecture, University of Maryland, College Park, MD 20742, USA; 4 Department of Agronomy and Horticulture, University of Nebraska Lincoln, Lincoln, Nebraska, NE 68583, USA; 5 National Center for Genome Resources, Santa Fe, NM 87505, USA

## Abstract

**Background:**

The Soybean Consensus Map 4.0 facilitated the anchoring of 95.6% of the soybean whole genome sequence developed by the Joint Genome Institute, Department of Energy, but its marker density was only sufficient to properly orient 66% of the sequence scaffolds. The discovery and genetic mapping of more single nucleotide polymorphism (SNP) markers were needed to anchor and orient the remaining genome sequence. To that end, next generation sequencing and high-throughput genotyping were combined to obtain a much higher resolution genetic map that could be used to anchor and orient most of the remaining sequence and to help validate the integrity of the existing scaffold builds.

**Results:**

A total of 7,108 to 25,047 predicted SNPs were discovered using a reduced representation library that was subsequently sequenced by the Illumina sequence-by-synthesis method on the clonal single molecule array platform. Using multiple SNP prediction methods, the validation rate of these SNPs ranged from 79% to 92.5%. A high resolution genetic map using 444 recombinant inbred lines was created with 1,790 SNP markers. Of the 1,790 mapped SNP markers, 1,240 markers had been selectively chosen to target existing unanchored or un-oriented sequence scaffolds, thereby increasing the amount of anchored sequence to 97%.

**Conclusion:**

We have demonstrated how next generation sequencing was combined with high-throughput SNP detection assays to quickly discover large numbers of SNPs. Those SNPs were then used to create a high resolution genetic map that assisted in the assembly of scaffolds from the 8× whole genome shotgun sequences into pseudomolecules corresponding to chromosomes of the organism.

## Background

The Department of Energy, Joint Genome Institute (JGI) has completed an 8× shotgun draft sequence of the soybean cultivar Williams 82 [1]. For initial assembly of the genome sequence, a preliminary 4× and 6.5× scaffold assembly was produced by the JGI, with the 6.5× assembly released to the public http://www.phytozome.net. This 6.5× assembly contained a total of 3,118 scaffolds totaling 993.5 Mb of sequence. Using the soybean Consensus Map 4.0, which contains a total of 5,500 markers [2], Schmutz et al., [1] associated a total of 296 of the 6.5× scaffolds with the genetic map. These scaffolds consisted of 949 Mb, or 95.6% of the total 6.5× assembly.

The initial assembly resulted in the anchoring of a large proportion of the genome to create 20 psuedomolecules corresponding with the 20 soybean chromosomes. However, it was subsequently evident that this initial psuedomolecule build had a significant number of assembly problems [1]. First and foremost was the insufficient resolution afforded by the Consensus Map 4.0, which had been constructed using five separate mapping populations and with most of the markers mapped in less than 100 individuals [2]. Second, many of the anchored scaffolds contained just one mapped marker, or contained multiple tightly linked markers whose map order was questionable due to insufficient recombination. Thus, proper orientation of those scaffolds was not possible or was questionable.

The ideal marker for anchoring and orienting the soybean genome is the single nucleotide polymorphism (SNP), primarily because SNPs are the most abundant marker available. Cultivated soybean [*Glycine max *(L.) Merr.] has nucleotide diversity (θ) of about 0.001 [3,4], which translates into an average SNP frequency of one SNP per 1000 bp of contiguous sequence. The wild ancestor *Glycine soja *(Sieb and Zucc.) has an estimated nucleotide diversity of θ = 0.00235, which is the equivalent of approximately one SNP per 425 bp [5]. Another advantage of SNPs is the wide array of currently available technologies for performing multiplex assays that can range from genotyping a few SNPs at a time to over 1 million SNPs in parallel [6]. One of these technologies is the GoldenGate assay, which can genotype 384 to 1,536 SNPs in 192 DNA samples in just three days. The reliability and rapidity of this assay was recently documented with soybean SNPs [2,7,8].

New high-throughput re-sequencing technologies have recently become available for generating greater amounts of DNA sequence quickly and inexpensively relative to standard Sanger sequencing [9]. Despite this advantage, large genomes still require a method to reduce genome complexity to a level that ensures accurate SNP discovery. One method utilizes high-throughput sequencing of the transcriptome through massively parallel pyro-sequencing technology [10]. While this method was successful, SNP discovery using this procedure is restricted to the expressed transcriptome and would likely not discover SNPs that could be used to anchor and orient non-coding DNA stretches of the genome.

The use of reduced representation libraries (RRLs) was first proposed in humans to efficiently find SNPs using Sanger sequencing [11]. A reduction in genome complexity is accomplished via the construction of an RRL with a restriction digestion followed by size selection. The use of fragments from a size-selected digestion permits a similar subset of fragments to be obtained from different genotypes that can be deep-sequenced for accurate SNP discovery. A procedure for high-throughput SNP discovery was recently described in cattle, and used an RRL combined with the sequence-by-synthesis (SBS) method on the clonal single molecule array (CSMA) platform manufactured by Illumina, Inc., with which short sequence reads could be compared to a reference genome for SNP discovery [12]. This approach successfully identified 62,042 putative SNPs. A subsequent analysis of 22,865 of these SNPs revealed a 91% validation rate, demonstrating the robustness of this SNP discovery method [12].

Our objective was to use the RRL approach with the SBS method on the CSMA platform from Illumina, Inc for the discovery of large numbers of soybean SNPs that could be developed into GoldenGate assays to create a new genetic map with higher resolution than Consensus Map 4.0 [2]. This high resolution genetic map would then help address the challenges faced in assembling and orienting the remainder of the soybean genome [1].

## Results

### Genome Analyzer sequence results

A total of 8,671,165 sequence reads of 33 bp in length were obtained for the *G. soja *accession PI 468916, yielding a total of more than 286 Mb of sequence (Table 1). More than 2,500 of the 33-mers, after being sequenced 300 or more times, were determined to be either repetitive nuclear DNA, or DNA from chloroplast or mitochondria. These were excluded from further analysis. From the PI 468916 sequence data, 1.96 million unique 33-mers were obtained, of which 1.82 million (92%) occurred five or fewer times. These PI 468916 short sequence reads were then aligned to the soybean, Williams 82, 6.5× scaffold assemblies for SNP discovery. A total of 4.21 million reads were ultimately aligned uniquely to the Williams 82, 6.5× scaffold assembly. The 4.21 million reads covered a total of 28 Mb of soybean sequence (after accounting for regions with multiple coverage). This translates into a 4.88× coverage of the 6.5× genome assembly for the purposes of SNP prediction.

**Table 1:** Numbers of occurrences of 33-mer Illumina Genome Analyzer sequence reads, numbers of 33-mers in each occurrence category as well as the total 33-mer reads and total bases in each occurrence category.

No. of occurrences of a particular 33-mer	No. of unique 33-mers	No. of 33 base reads	Total bases
500 plus	1,293	2,142,203	70,692,699
300-500	1,414	536,701	17,711,133
100-299	6,561	1,097,771	36,226,443
35-99	15,119	871,270	28,751,910
20-34	14,510	374,818	12,368,994
15-20	12,040	206,542	6,815,886
11-14	15,645	192,046	6,337,518
9-10	15,234	143,581	4,738,173
7-8	29,215	216,367	7,140,111
6	26,648	159,888	5,276,304
5	43,105	215,525	7,112,325
4	72,955	291,820	9,630,060
3	130,555	391,665	12,924,945
2	259,225	518,450	17,108,850
1	1,312,518	1,312,518	43,313,094

**TOTAL**	**1,956,037**	**8,671,165**	**286,148,445**

### SNP Prediction and Validation

Multiple methods were initially tested for SNP prediction and filtering. We used the GMAP [13] and Maq [14] mapping software. The GMAP software directly compares predicted bases in short-read query and genomic reference sequences, while the Maq software additionally uses quality scores in the short-read sequences, and provides additional output information about the consensus quality of SNP calls. We made three validation runs, on a preliminary 4× scaffold assembly; and one "production" run on the preliminary 6.5× scaffold assembly. The validation runs used GMAP with multiple short sequence reads to predict SNPs, and Maq with either multiple or single short sequence reads to predict SNPs. The production run used predominantly multiple short sequence reads to predict SNPs, with single short sequence read predicted SNPs where necessary in areas of particular interest. Because only one of the programs uses quality scores, the parameter sets are not trivially comparable. However, both programs were tunable for these objectives. For each of the tested methods, we picked parameter sets with the general goals of predicting unique, polymorphic, well-supported SNPs, given constraints of available sequence and the need to generate markers in certain marker-poor regions.

The first SNP prediction method, for testing and validation involved the use of GMAP software [13] for alignment of unique PI 468916 short sequence reads to the Williams 82, preliminary 4× scaffold assembly, with SNPs predicted from the alignments. The preliminary 4× scaffold assembly was a "test run" produced by the sequencing consortium for an initial assessment of assembly characteristics of the genome. At the same time, we used this early assembly to initially test and validate using next generation sequencing and different software for SNP-prediction. The GMAP alignment method, with stringent match criteria (using only high-quality reads, unique mappings, multiple-reads SNP support; details in Materials and Methods), produced a total of 10,778 predicted SNPs. The validation set for the GMAP method consisted of 635 primer pairs designed to flank each candidate SNP. Of these 635 primer pairs, a total of 535 produced a sequence tagged site with high quality sequence surrounding the predicted SNP, and 456 of the 535 produced amplicons containing the predicted SNP, which constituted an 85% validation rate.

The second SNP prediction method employed was the Maq mapping and assembly software [14] to align unique PI 468916 short sequence reads to the preliminary 4× scaffold assembly and predict SNPs from that assembly. The Maq analysis method, when used with selected parameters (minimum consensus-base quality of 20, unique read placement; details in Materials and Methods), produced a total of 25,047 predicted SNPs, each predicted using one or more short sequence reads aligned to one position within the genome. The first validation set for the Maq procedure consisted of 48 primer pairs designed to flank SNPs predicted from two or more short sequence reads. Of these 48 primer pairs, a total of 40 produced a sequenced tagged site (STS) with high quality sequence surrounding the predicted SNP, and 37 of the 40 produced amplicons containing the predicted SNP, which translated into a 92.5% validation rate.

We also tested the validation rate of predicting SNPs with a single short read. For this test, the Maq analysis method above was used, but without the requirement for multiple read support of predicted SNPs. A total of 48 primer pairs were designed to flank SNPs predicted from only one short sequence read. Of the 48 primer pairs tested, 43 produced an STS with high quality sequence surrounding the predicted SNP, and 34 of those 43 produced amplicons that contained the predicted SNP, which resulted in a 79% validation rate.

To identify SNPs best suited for GoldenGate assay design and for anchoring and orienting the preliminary 6.5× scaffold assembly, we used a modification of the Maq method described in the validation tests above. In this "production" run, we required a consensus score of at least 27, and that the flanking 120 bases be at least 2/3 non-repetitive sequence (see Materials and Methods for implementation). Because of the high (79%) validation rate for SNPs called from single short sequence reads using Maq, these SNPs were also included in this dataset so that additional markers on more of the smaller unmapped scaffolds could be anchored to the genetic map. In total, 7,108 SNPs were predicted for use in anchoring and orienting additional scaffolds. Ultimately, 1,536 SNPs were selected from this pool of 7,108 SNPs to create the higher resolution map needed for validation of scaffold ends, for anchoring of additional scaffolds, and for mitigating the ambiguity of orientations of anchored scaffolds - all of which required a map with markers placed in the existing gaps that are present within the soybean Consensus Map 4.0. These 1,536 SNPs were used to create an Illumina GoldenGate **soy**bean **o**ligo **p**ool **a**ll (SoyOPA-4) [Additional file 1]. The SoyOPA-4 produced 1,254 successful GoldenGate assays indicating that the predicted SNPs had a validation and assay conversion rate of 81.6%.

### High Resolution Genetic Map

SoyOPA-4 was used to genotype 470 F_5_-derived RILs from the Williams 82 × PI 468916 (W82 × 468) population with the 1,254 polymorphic SNPs. A total of 26 of the 470 RILs were excluded from further analysis due to marker heterozygosity levels > 20%, which suggested that those 26 RILs trace to outcrosses occurring during generation advance, rather than being true F_5_-derived lines. To tie the newly constructed high resolution genetic map to the existing Soybean Consensus Map 4.0, SoyOPA-3 was used to genotype a subset of 282 RILs from the W82 × 468 population. From these 282 genotyped RILs, the genotype data of 14 RILs were subsequently eliminated which were in common with the 26 RILs that had already been eliminated due to high heterozygosity levels. SoyOPA-3 was one of three previously designed custom soybean OPAs developed and tested by Hyten et al. [2,7]. SoyOPA-3 contains 1,396 SNPs that had been mapped on the Soybean Consensus Map 4.0 and had been developed so that all SNPs included in the OPA were polymorphic within at least one of three RIL mapping populations used in the creation of the Soybean Consensus Map 4.0 [2]. Of the 1,396 SNPs on SoyOPA-3 that mapped to the Soybean Consensus Map 4.0, a total of 565 were polymorphic between Williams 82 and PI 468916.

A total of 550 of the 565 SNPs from SoyOPA-3 and 1,240 of the 1,254 SNPs from SoyOPA-4 were mapped using 444 RILs to create the 20 linkage groups that correspond to the 20 chromosomes of soybean and had an estimated total genetic length of 2,537 cM. The remaining 29 SNPs were not linked to any of the 20 linkage groups. The average level of heterozygosity observed in the population was 6.3% which is the expected level of heterozygosity for a RIL population in the F_5 _generation. Segregation distortion was observed for multiple tightly linked markers in 16 regions throughout the genome [Additional file 2].

To determine if the mapping of SoyOPA-3 on a subset of the 444 RILs caused any significant expansions or contractions of the genetic map, a separate map with only the SNPs from SoyOPA-4 was created [Additional file 2]. Comparing the map created using only 1,240 SoyOPA-4 SNPs to the map created using all 1,790 SNPs from SoyOPA-3 and SoyOPA-4 revealed only one substantive change of 22 cM on chromosome 5. This change was due to the elimination of the SoyOPA-3 SNPs leaving a gap of 66 cM between adjacent SoyOPA-4 SNPs. There were a total of 16 discrepancies between the two maps that were 2 to 10 cM with all other discrepancies between the two maps being less than 2 cM in genetic distances between the SoyOPA-4 SNP markers.

### Anchoring of the Soybean Genome

The high-resolution W82 × 468 genetic map with 1,790 SNP markers was successful in anchoring and orienting additional scaffolds in the subsequent 8× scaffold assembly using the preliminary 6.5× scaffold assembly for initial SNP discovery and assay development [1]. It added markers to 335 8× assembly scaffolds, of which 23 scaffolds (totaling 7.1 Mb) were previously unmapped in the preliminary 6.5× scaffold assembly. The high-resolution map also oriented 151 scaffolds as a result of positioning markers such that there was at least 1 cM between the most widely separated markers on any given scaffold. The map also helped to evaluate the integrities of the continuous sequence of the scaffolds by adding markers to regions of scaffolds that were previously without markers, and to scaffold ends [1].

## Discussion

The use of a reduced representation library, coupled with next generation sequencing and the initial release of soybean genome sequence [1], provided a powerful method for the additional discovery of large numbers of SNPs. The validation rate of predicted SNPs varied from 79% up to 92.5%. Not surprisingly, the largest factor for this range in validation was due to the use of one versus multiple short reads to predict a SNP. The decrease in the validation rate arising from the use of only one read for SNP prediction, though modest in percentage terms, may be due to sequencing error from the Genome Analyzer. Still, the validation rate of 79% for SNPs predicted from a single short sequence read was sufficiently high to lead us to ultimately use 63 SNPs predicted by a single short sequence read to serve as markers in regions that otherwise might have not been anchored or properly oriented in the 8× Glyma1.01 assembly.

The production run Maq analysis method resulted in the conversion of 81.6% of the predicted SNPs into working GoldenGate assays. Hyten et al. [7] demonstrated that with high confidence SNPs, the conversion rate in soybean for developing a successful GoldenGate assay from a validated SNP was 89%. Taking into account this 89% conversion rate, the production run Maq analysis method effectively had a SNP validation rate of 91.7%. This is very similar to the 92.5% validation rate obtained from the initial Maq analysis method based upon two or more reads. This high validation rate matching the validation rate obtained with two or more reads was expected to some degree, given that only 63 of the 1,536 SNPs were predicted based upon a single short sequence read.

Using the production run Maq assembly, we were able to target 1,536 SNPs (of the total 7,108 SNPs) onto the preliminary 6.5× scaffold assemblies to help improve the subsequent anchoring and orientation of the 8× scaffold assembly of soybean into the 20 pseudomolecule Glyma1.01 build [1]. We were able to target SNPs to scaffolds that heretofore could not be anchored using the Consensus Map 4.0. We were able to orient additional scaffolds. Where possible, two markers were chosen at the ends of each unanchored or un-oriented scaffold. This led to the W82 × 468 genetic map having an uneven distribution of markers as they were clustered in regions where the Consensus Map 4.0 had few or no markers [Additional file 3]. Overall the initial use of the Consensus Map 4.0 followed by the subsequent use of the W82 × 468 high-resolution map, resulted in a whole genome sequence of soybean of which more than 97% is anchored to the genetic map [1]. The high resolution map ultimately provided markers for 335 of the 8× scaffolds which totaled more than 872 Mb of the completed genome sequence. Many of these scaffolds contain markers from the Consensus Map 4.0, but the high-resolution map provided not only a rigorous confirmation of scaffold contiguity, but also ordered and oriented 23 of the 8× scaffolds for which no markers existed on the consensus map.

## Conclusions

The availability of a diversity of both low- and high-multiplex SNP assay methods makes SNPs an ideal marker for QTL mapping, association analysis, marker-assisted selection (MAS), and the construction of high density genetic maps for fine-mapping and cloning of agronomically important genes. We have demonstrated how next generation sequencing combined with high-throughput SNP detection assays can quickly discover a large number of SNPs that can then be used to create a high resolution genetic map. Further, such high-resolution maps are critical for accurate placement of sequence scaffolds into chromosomal pseudomolecules.

## Methods

### Reduced Representation Library Construction

Seeds of the *G. soja *genotype PI 468916 were obtained from the USDA-ARS, Soybean Germplasm Collection courtesy of Dr. Randall L. Nelson. DNA was isolated from bulk leaf tissue of 10 to 15 plants as described by Keim et al. (1988). Four different blunt end restriction enzyme combinations each containing five different restriction enzymes each were tested as an effort to reduce the likelihood of repetitive sequence. A single combination of five enzymes resulting in the least banding in the 100 to 150 bp region of the restriction digest was selected. The restriction digestion of PI 468916 to create the RRL consisted of digesting a total of 50 μg of DNA with the combination of 30 units of *Hae*III, 15 units of *Psi*I, 15 units of *Ssp*I, 30 units of *Rsa*I, and 15 units of *Msl*I. The restriction digestion was carried out overnight at 37°. The digested DNA was then run on a 2% agarose gel and the digestion products were excised from the gel in the 100 to 150 bp region. The QIAquick Gel Extraction Kit (Qiagen, Hilden, Germany) was used as per the manufacturer's protocol to obtain a total of 4,300 ng of size selected DNA. The blunt-ended DNA fragments were provided to Illumina for sequencing on the Genome Analyzer (Illumina, Inc; San Diego, CA, USA). The sequence data obtained from Illumina contained 33 bp sequence tags in which every base is given a quality score that is similar to a Phred score and has a maximum value of 40 [12]. The Illumina sequence data have been deposited in the NCBI, Sequence Read Archive [GenBank:SRA010205].

### SNP discovery

Initially the GMAP method was used for SNP discovery from the sequence produced from the reduced representation library with the Illumina Genome Analyzer. GMAP software [13] was used for alignment of the 33 bp PI 468916 Illumina Genome Analyzer reads with the Williams 82 preliminary 4× scaffold assembly for the discovery of putative SNPs. The following constraints were used in the filtering for the identification of SNPs: 1) short reads which contained a base with a quality score < 10 were eliminated, 2) reads were selected that had only one alignment to the Williams 82 reference sequence and had to have 32 or 33 matching nucleotides with no part of the sequence part of a repeat (as determined by GMAP, in the 3^rd ^column of the BLAT-output PSL format), no "N" bases in the alignment, and no gaps present in the query sequence and no gaps in the reference sequence, 3) alignments that had any conflicting nucleotides were eliminated, 4) SNPs where both flanking 25 nt regions were repetitive were eliminated (specifically, when both flanking regions matched other locations in the genome with two or fewer mismatches), 5) SNPs had to be supported by at least two short sequence reads, 6) filter out any remaining sequence containing an N. In addition, Genome Analyzer sequence reads with matches to chloroplast sequence [GenBank:NC_007942] or *Nicotiana *mitochondrion sequence [GenBank:NC_006581] were discarded.

While the above work was in progress, the Maq mapping and assembly software [14] was tested as an alternative method to GMAP for alignment of Illumina Genome Analyzer sequence reads with the Williams 82 preliminary 4× scaffold assembly and later when the preliminary 6.5× scaffold assembly became available for SNP discovery. Three rounds of SNP predictions were made using Maq: one for **v**alidation using **m**ultiply-**s**upported SNPs (VMS); one for **v**alidation using **s**ingly-**s**upported SNPs (VSS); and a production run. The runs for validation used the same filtering steps described below for the production run, except where noted. The following constraints were used in the filtering for the identification of putative SNPs using Maq software: 1) Occurrences of two or more SNPs within a 25-base window were eliminated, 2) SNPs with ambiguous consensus bases (i.e., bases other than A, C, T or G) were eliminated, 3) a minimum consensus-base quality score of 20 for VMS and VSS (27 for the production run) was required, 4) an average read-copy of 1.00 i.e., the SNP maps to one place in the Williams 82 reference genome, 5) a minimum Maq mapping score of 30 was required, and 6) with VMS a minimum of two short sequence reads were required to support each putative SNP. In addition, Genome Analyzer reads with matches to chloroplast sequence [GenBank:NC_007942] or *Nicotiana *mitochondrion sequence [GenBank:NC_006581] were discarded. Additionally, we removed SNPs from repetitive regions, using the following protocols. For VMS and VSS, extract the 121 nt region centered on the SNP and exclude the SNP if this region contained an "N". If either 25-mer flanking a SNP hit any other place in the genome with at most two mismatches using an ungapped BLAST it was considered repetitive; if both were repetitive, the SNP was excluded. For the production run, extract a 601 nt region centered on the SNP and exclude the SNP if this region contained an "N". If any 25-mer in the 300 nt up- or downstream region hit five or more other positions in the genome with at most two mismatches using an ungapped BLAST, it was considered repetitive; SNPs whose flanking regions were more than 1/3 repetitive were also excluded.

### SNP Validation through Resequencing

Based upon the underlying Williams 82 preliminary 4× scaffold assembly, polymerase chain reaction (PCR) primers were designed using Primer 3 software [15]. The primers were designed to the flanking sequence of 620 putative SNPs for the GMAP method and 96 putative SNPs for the Maq method. Electronic PCR [16] was used to select primer pairs that, based upon the Williams 82 preliminary 4× scaffold assembly, would be anticipated to be locus specific. The predicted amplicon lengths ranged from approximately 100 to 700 bp in length. Initial amplification, sequence analysis and alignment for validation of putative SNPs between Williams 82 and PI 468916 were performed as described by Choi et al. [3].

### Population Development

Four hundred and seventy F_5_-derived recombinant inbred lines (RIL) were created at the Beltsville Agricultural Research Center, Beltsville, MD, USA, from a cross made in 2004 between the soybean cultivar Williams 82 [17] and the wild soybean PI 468916 followed by plant-to-row descent. A total of 2,000 F_2 _seeds were planted in the field at Beltsville, MD the summer of 2005. Approximately 10 to 15 F_3 _seeds were harvested from each of 1,690 individual F_2 _plants. In the summer of 2006, five F_3 _seeds harvested from each of 1,000 randomly selected F_2 _plants were planted in hill plots and subsequently thinned to one F_3 _plant two weeks after planting. A total of 10 to 15 F_4 _seeds were harvested from each of 932 F_3 _plants. In the winter of 2006-2007, three F_4 _seeds from 600 randomly selected F_3 _plants were planted in the greenhouse for the next generation advance and subsequently thinned to one plant after two weeks. Ten to fifteen F_5 _seeds were harvested from each of 583 F_4 _plants. In the summer of 2007, five F_5 _seeds from each of the 583 F_4 _plants were planted in hill plots and thinned to one F_5 _plant two weeks after planting. DNA was isolated from 470 RILs randomly selected from the 583 F_5 _plants by taking leaf tissue from the single F_5 _plant from which the RIL was derived. The Qiagen DNeasy 96 Plant DNA extraction kit (Qiagen, Hilden, Germany) as per the manufacturer's protocol was used to obtain purified DNA for the GoldenGate Assay analysis. All seeds were harvested from each F_5 _plant to create an F_5_-derived line. To help minimize segregation distortion in creating the population, seeds in all generations were harvested as soon as individual plants became mature to avoid loss of seeds due to shattering.

### GoldenGate assay and Genetic Map Development

A total of 1,536 SNPs were selected to develop a 1,536 GoldenGate assay [Additional file 1]. The SNPs were selected from scaffolds of the Williams 82 preliminary 6.5× scaffold assembly to help validate scaffold ends, anchor additional scaffolds, and improve orientations of anchored scaffolds. Of the 1,536 SNPs selected, 242 SNPs were selected to anchor scaffolds not previously anchored with the Consensus Map 4.0, 227 SNPs were selected to orient scaffolds with too little marker separation from the Consensus Map 4.0, 217 SNPs were selected to internal marker-poor islands (regions greater than 2 Mb without markers), and the remainder were selected near scaffold ends to help assess scaffold integrity. The 1,536 SNPs were selected from SNPs predicted using the production run Maq analysis method described previously. In the case of 63 SNPs, the SNP discovery protocol was relaxed to allow SNPs to be called with a single short sequence read in an effort to target markers to more of the smaller, harder-to-map scaffolds.

For GoldenGate assay design all SNPs were required to have a designability rank score >0.4, which is predictive of a moderate to high success rate for the conversion of the SNP into a working GoldenGate assay. The 1,536 chosen SNPs made up the custom Oligo Pool All (OPA) which was given the name SoyOPA-4. To help anchor and align the genetic map created by the SNPs on SoyOPA-4, the previously designed and tested SoyOPA-3 [2] was tested on 282 RILs of the Williams 82 × PI 468916 population per the manufacturer's protocol and as described by Fan et al. [18] and Hyten et al. [7]. SoyOPA-4 was tested on 470 RILs per the manufacturer's protocol and as described by Fan et al. [18] and Hyten et al. [7]. The Illumina BeadStation 500G (Illumina Inc., San Diego, CA) was used for genotyping the GoldenGate assay. The automatic allele calling for each locus and the calculation of minor allele frequencies were accomplished with the GenCall software (Illumina Inc., San Diego, CA). All GenCall data were visually inspected and re-scored if any errors in calling the homozygous or heterozygous clusters were evident.

JoinMap 3.0 software [19] was used for creation of the high resolution genetic maps. Genotype data for 26 RILs were excluded from the full genotype data set due to high heterozygosity levels detected when genotyped with SoyOPA-3 and SoyOPA-4. The exclusion of these 26 RILs left genotype data for 268 RILs genotyped with SoyOPA-3 and 444 RILs genotyped with SoyOPA-4 to create the high resolution genetic map. Genetic distances were calculated using the Kosambi mapping function. A minimum LOD ≥ 10.0 and a maximum distance ≤ 50 cM were used to test linkages among markers.

## Authors' contributions

DLH designed and oversaw the study, developed the mapping population, designed the GoldenGate OPA, performed genotyping analysis of the GoldenGate assay, and drafted the manuscript. SBC, NW and QS performed the SNP discovery, designed the GoldenGate OPA, and performed additional analysis of the short read data. SBC also assisted in preparing the manuscript. QS created the high resolution linkage map. ECF created the reduced representation library, performed sequencing of the validation sets, helped to develop the mapping population, and assisted in preparation of the manuscript. RCS helped conceive the study. JES assisted in the analysis of the genetic map data and in preparing the manuscript. ADF and GDM assisted in an initial analysis of the genome analyzer short reads. PBC designed and oversaw the study, developed the mapping population, and assisted in preparing the manuscript. All authors read and approved the final manuscript.

## Supplementary Material

Additional file 1 **SoyOPA-4 SNPs**. The 1,536 SNPs along with the flanking sequence to design the GoldenGate assay SoyOPA-4. Click here for file

Additional file 2 **Williams 82 × PI 468916 genetic map**. The high-resolution Williams 82 × PI 468916 genetic map created with 1,790 SNPs genotyped on 268 or 444 recombinant inbred lines (RILs). Also shown is the high-resolution map resulting when only using the 1,254 SNPs genotyped with SoyOPA-4 on 444 RILs and the Consensus Map 4.0 position for any SNPs in common between the two genetic maps. Click here for file

Additional file 3 **Williams 82 × PI 468916 comparison to the soybean Consensus Map 4.0**. The charts of the 20 linkage groups from the Consensus Map 4.0 (left chart) compared to the same 20 linkage groups produced from the 444 recombinant inbred lines of Williams 82 × PI 468916 (right chart). Click here for file

## References

[B1] Schmutz J Cannon SB Schlueter J Ma J Mitros T Nelson W Hyten DL Song Q Thelen JJ Cheng J Genome seqeunce of the paleopolyploid soybean Nature 2010 463 178 183 10.1038/nature08670 20075913

[B2] Hyten DL Choi I-Y Song Q Specht JE Carter TE Shoemaker RC Hwang E-Y Matukumalli LK Cregan PB A high density integrated genetic linkage map of soybean and the development of a 1,536 Universal Soy Linkage Panel for QTL mapping Crop Sci 2010 in press

[B3] Choi I-Y Hyten DL Matukumalli LK Song Q Chaky JM Quigley CV Chase K Lark KG Reiter RS Yoon M-S A soybean transcript map: gene distribution, haplotype and single-nucleotide polymorphism analysis Genetics 2007 176 1 685 696 10.1534/genetics.107.070821 17339218 PMC1893076

[B4] Zhu YL Song QJ Hyten DL Van Tassell CP Matukumalli LK Grimm DR Hyatt SM Fickus EW Young ND Cregan PB Single-nucleotide polymorphisms in soybean Genetics 2003 163 3 1123 1134 12663549 10.1093/genetics/163.3.1123 PMC1462490

[B5] Hyten DL Song Q Zhu Y Choi IY Nelson RL Costa JM Specht JE Shoemaker RC Cregan PB Impacts of genetic bottlenecks on soybean genome diversity Proc Natl Acad Sci USA 2006 103 45 16666 16671 10.1073/pnas.0604379103 17068128 PMC1624862

[B6] Fan JB Chee MS Gunderson KL Highly parallel genomic assays Nat Rev Genet 2006 7 8 632 644 10.1038/nrg1901 16847463

[B7] Hyten DL Song Q Choi IY Yoon MS Specht JE Matukumalli LK Nelson RL Shoemaker RC Young ND Cregan PB High-throughput genotyping with the GoldenGate assay in the complex genome of soybean Theor appl genet 2008 116 7 945 952 10.1007/s00122-008-0726-2 18278477

[B8] Hyten DL Smith JR Frederick RD Tucker ML Song Q Cregan PB Bulked Segregant Analysis Using the GoldenGate Assay to Locate the Rpp3 Locus that Confers Resistance to Soybean Rust in Soybean Crop Sci 2009 49 1 265 271 10.2135/cropsci2008.08.0511

[B9] Metzker ML Emerging technologies in DNA sequencing Genome Res 2005 15 12 1767 1776 10.1101/gr.3770505 16339375

[B10] Barbazuk WB Emrich SJ Chen HD Li L Schnable PS SNP discovery via 454 transcriptome sequencing Plant J 2007 51 5 910 918 10.1111/j.1365-313X.2007.03193.x 17662031 PMC2169515

[B11] Altshuler D Pollara VJ Cowles CR Van Etten WJ Baldwin J Linton L Lander ES An SNP map of the human genome generated by reduced representation shotgun sequencing Nature 2000 407 6803 513 516 10.1038/35035083 11029002

[B12] Van Tassell CP Smith TP Matukumalli LK Taylor JF Schnabel RD Lawley CT Haudenschild CD Moore SS Warren WC Sonstegard TS SNP discovery and allele frequency estimation by deep sequencing of reduced representation libraries Nat Methods 2008 5 3 247 252 10.1038/nmeth.1185 18297082

[B13] Wu TD Watanabe CK GMAP: a genomic mapping and alignment program for mRNA and EST sequences Bioinformatics 2005 21 9 1859 1875 10.1093/bioinformatics/bti310 15728110

[B14] Li H Ruan J Durbin R Mapping short DNA sequencing reads and calling variants using mapping quality scores Genome Res 2008 18 11 1851 1858 10.1101/gr.078212.108 18714091 PMC2577856

[B15] Rozen S Skaletsky H Primer3 on the WWW for general users and for biologist programmers Methods Mol Biol 2000 132 365 386 10547847 10.1385/1-59259-192-2:365

[B16] Schuler GD Sequence mapping by electronic PCR Genome Res 1997 7 5 541 550 9149949 10.1101/gr.7.5.541 PMC310656

[B17] Bernard RL Cremeens CR Registration of 'Williams 82' soybean Crop Sci 1988 28 6 1027 1028

[B18] Fan JB Oliphant A Shen R Kermani BG Garcia F Gunderson KL Hansen M Steemers F Butler SL Deloukas P Highly parallel SNP genotyping Cold Spring Harb Symp Quant Biol 2003 68 69 78 10.1101/sqb.2003.68.69 15338605

[B19] Van Ooijen JW Voorrips RE JoinMap 3.0 software for the calculation of genetic linkage maps 2001 Plant Research Internation, Wageningen, the Netherlands

