# Supplementary material for: High-throughput SNP discovery through deep resequencing of a reduced representation library to anchor and orient scaffolds in the soybean whole genome sequence
Source: BMC Genomics. 2010 Jan 15;11:38. doi: 10.1186/1471-2164-11-38 (PMC2817691; doi:10.1186/1471-2164-11-38)
Supplement: Additional file 3 — Williams 82 × PI 468916 comparison to the soybean Consensus Map 4.0. The charts of the 20 linkage groups from the Consensus Map 4.0 (left chart) compared to the same 20 linkage groups produced from the 444 recombinant inbred lines of Williams 82 × PI 468916 (right chart). [file 1471-2164-11-38-S3.PPT]

## Slide 1
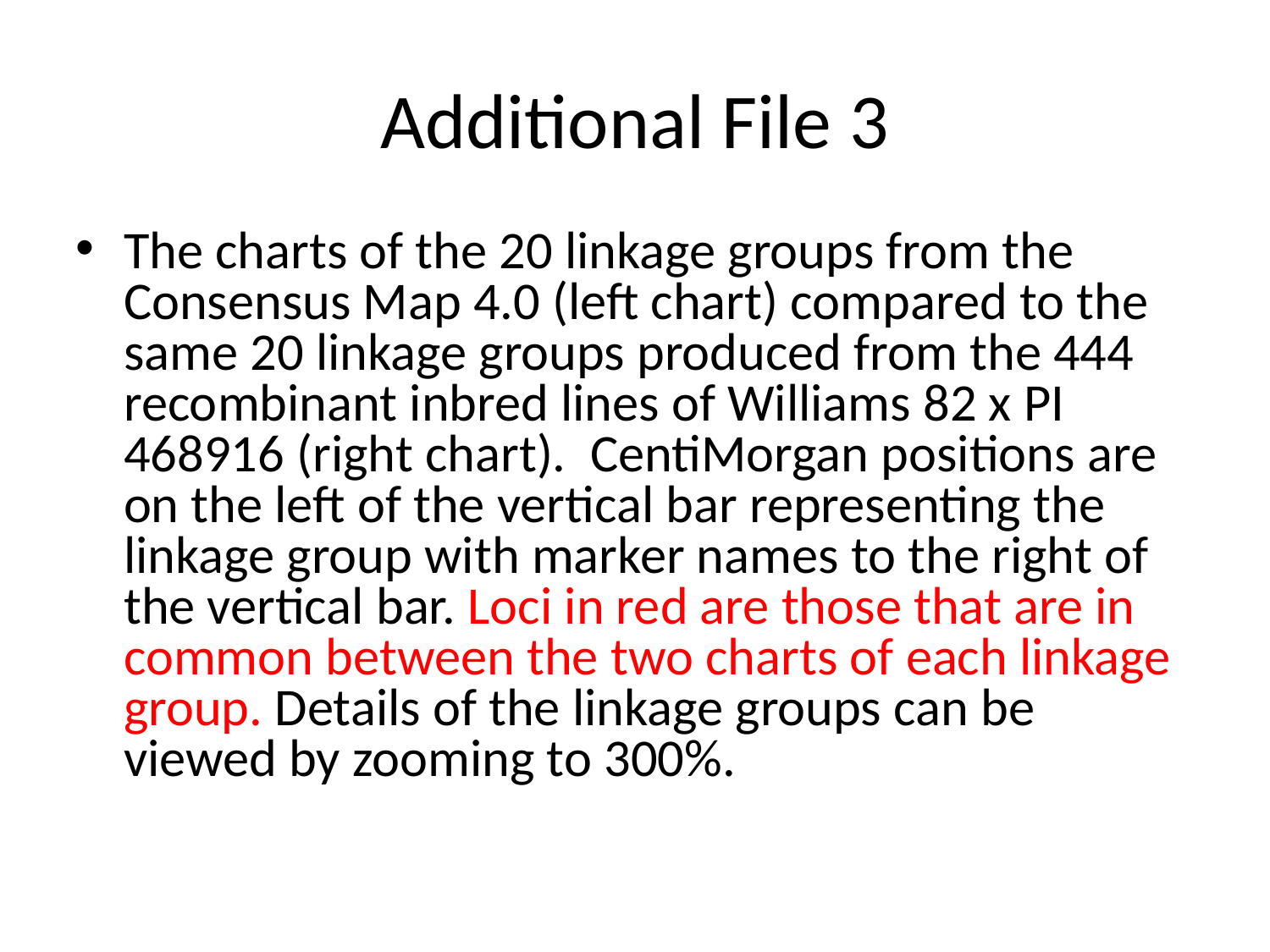

# Additional File 3
The charts of the 20 linkage groups from the Consensus Map 4.0 (left chart) compared to the same 20 linkage groups produced from the 444 recombinant inbred lines of Williams 82 x PI 468916 (right chart). CentiMorgan positions are on the left of the vertical bar representing the linkage group with marker names to the right of the vertical bar. Loci in red are those that are in common between the two charts of each linkage group. Details of the linkage groups can be viewed by zooming to 300%.

## Slide 2
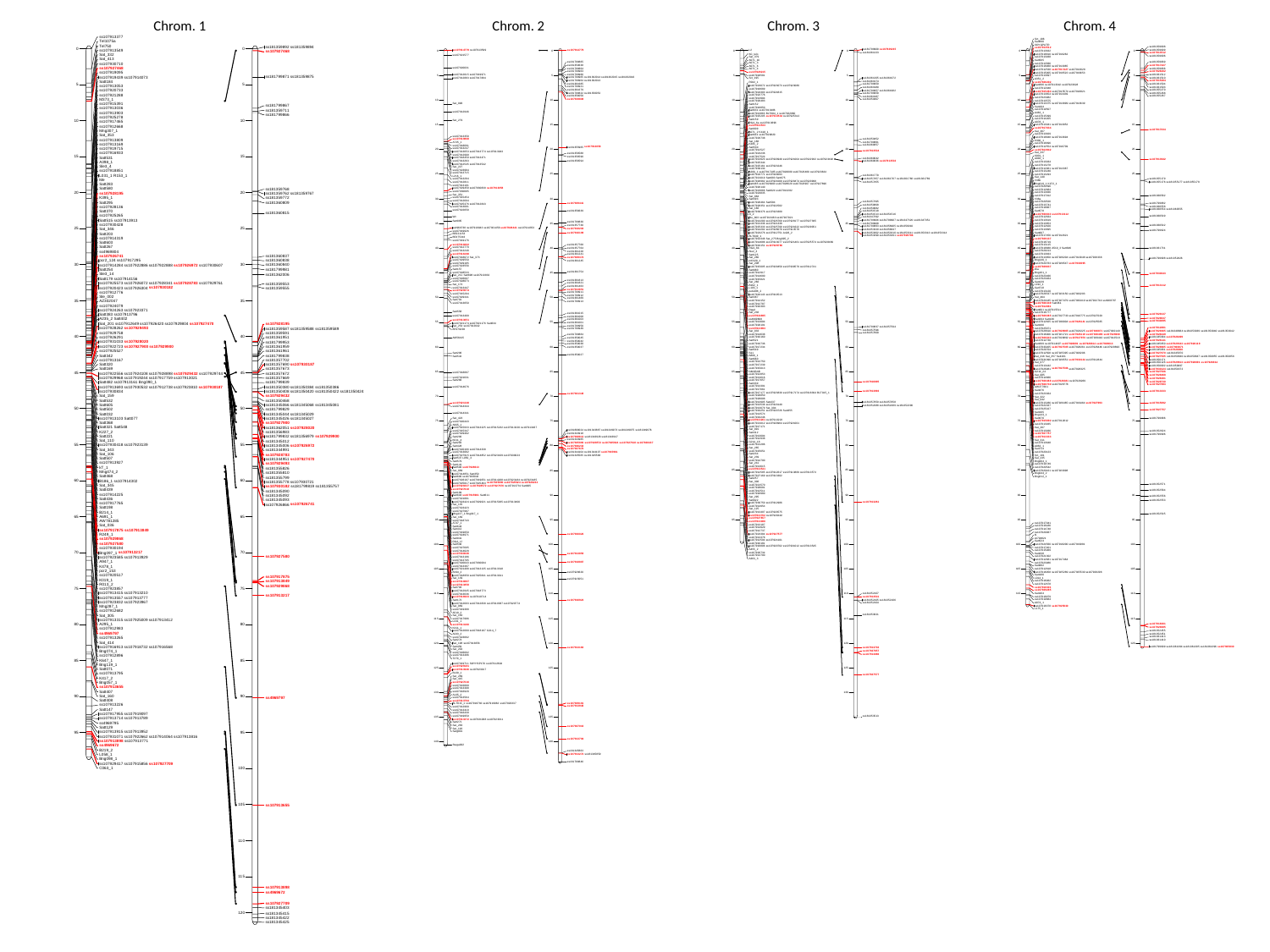

Chrom. 1
Chrom. 2
Chrom. 3
Chrom. 4

## Slide 3
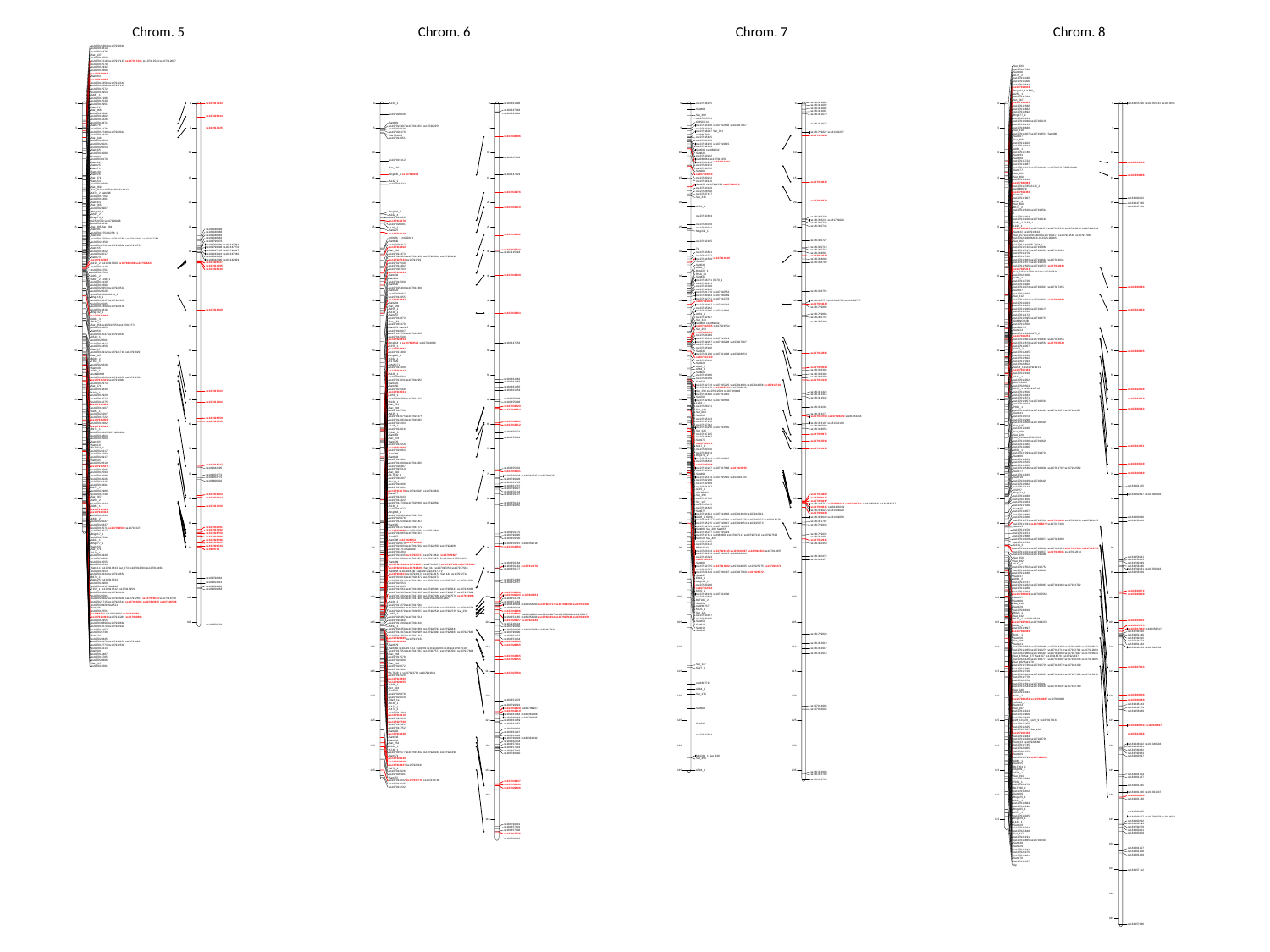

Chrom. 5
Chrom. 6
Chrom. 7
Chrom. 8

## Slide 4
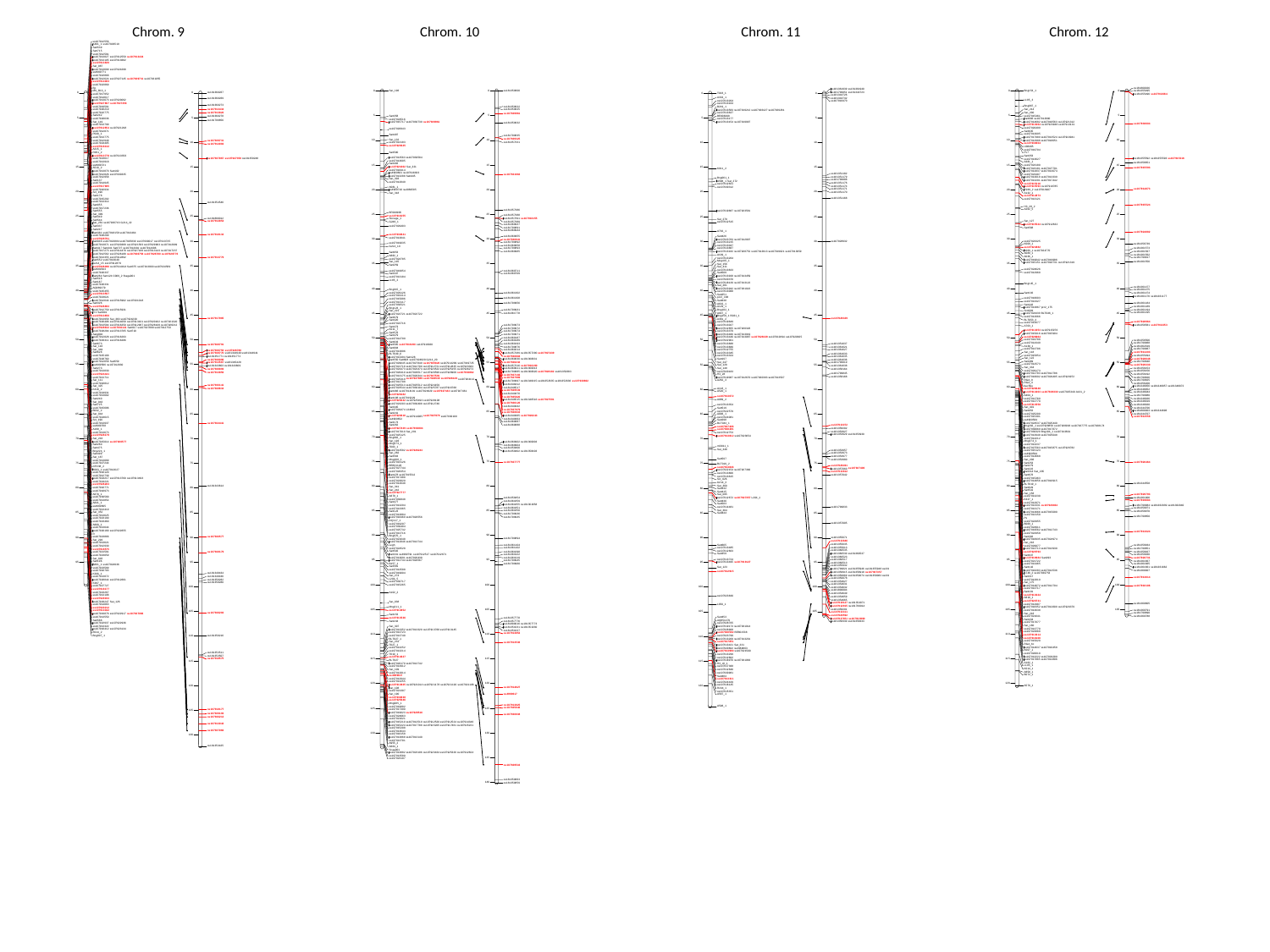

Chrom. 9
Chrom. 10
Chrom. 11
Chrom. 12

## Slide 5
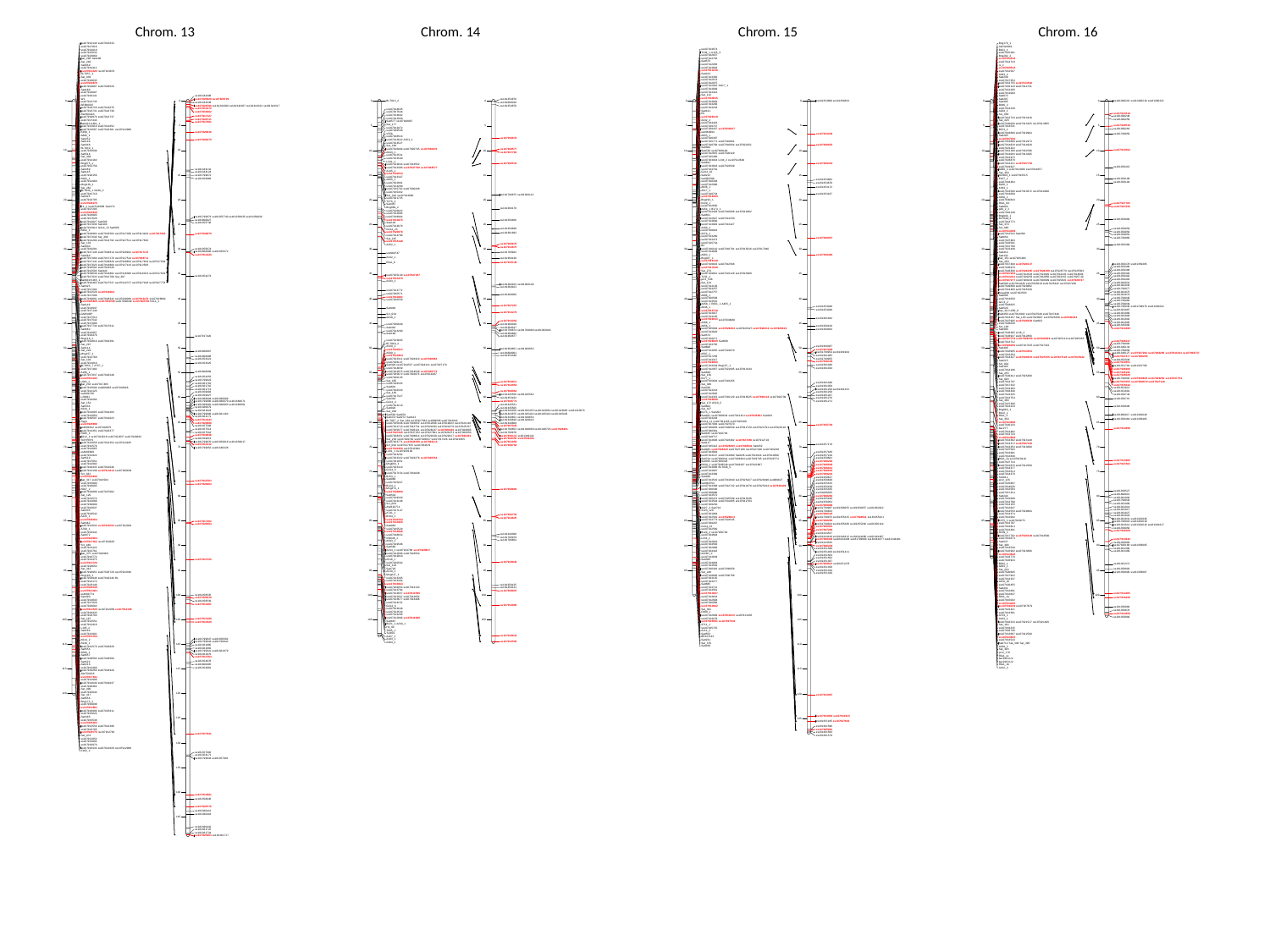

Chrom. 13
Chrom. 14
Chrom. 15
Chrom. 16

## Slide 6
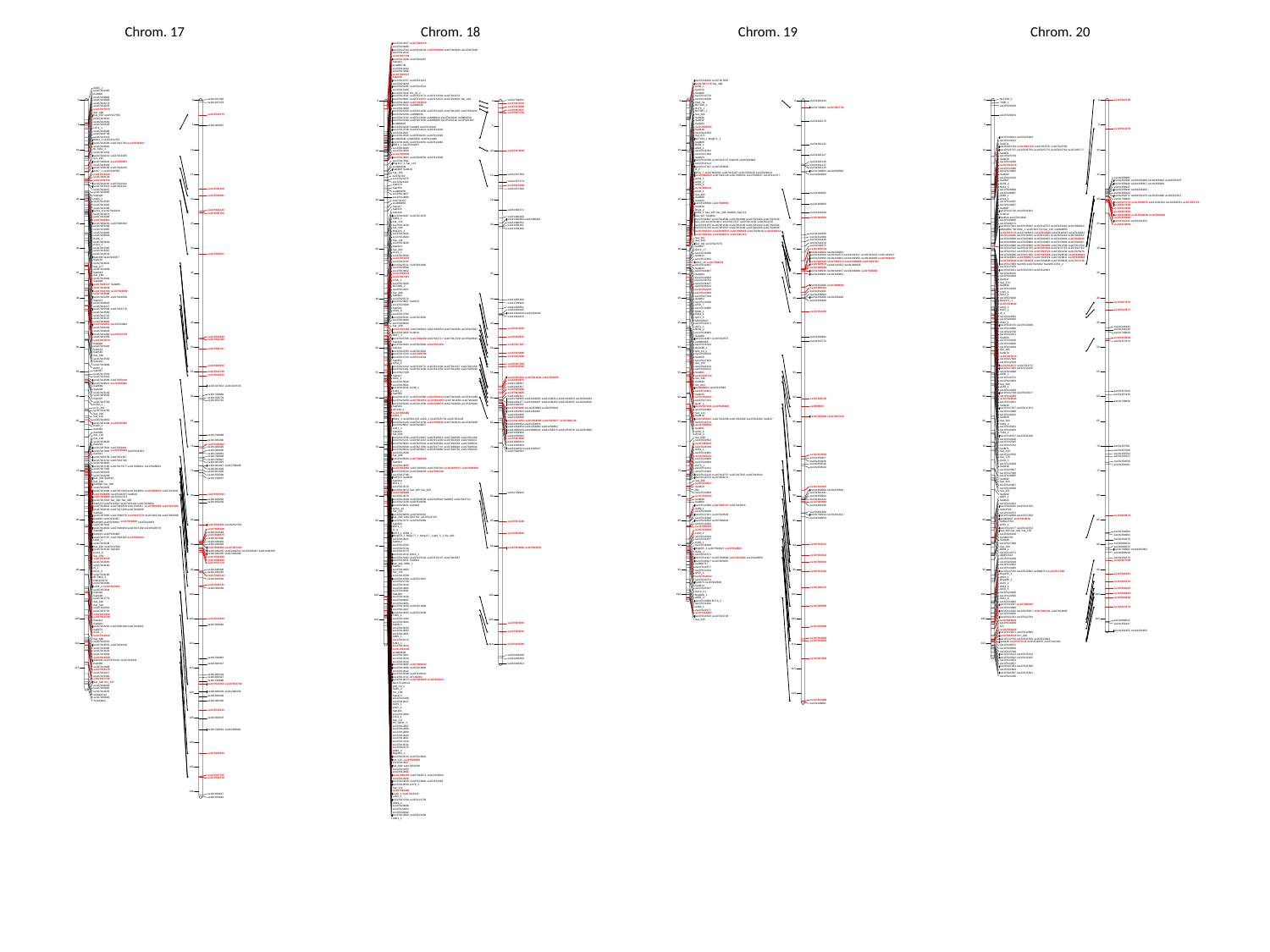

Chrom. 17
Chrom. 18
Chrom. 19
Chrom. 20
